# A Critical Assessment of the Equal-Environment Assumption of the Twin Method for Schizophrenia

**DOI:** 10.3389/fpsyt.2015.00062

**Published:** 2015-04-28

**Authors:** Roar Fosse, Jay Joseph, Ken Richardson

**Affiliations:** ^1^Division of Mental Health and Addiction, Vestre Viken Hospital Trust, Lier, Norway; ^2^Clinical Psychologist in Private Practice, Oakland, CA, USA; ^3^Independent Researcher/Formerly Open University UK, Durham, UK

**Keywords:** classical twin method, schizophrenia, equal-environment assumption, social adversities, intraclass correlations

## Abstract

The classical twin method (CTM) is central to the view that schizophrenia is ~80% heritable. The CTM rests on the equal-environment assumption (EEA) that identical and fraternal twin pairs experience equivalent trait-relevant environmental exposures. The EEA has not been directly tested for schizophrenia with measures of child social adversity, which is particularly etiologically relevant to the disorder. However, if child social adversity is more similar in identical than fraternal pairs in the general twin population, the EEA is unlikely to be valid for schizophrenia, a question which we tested in this study. Using results from prior twin studies, we tested if intraclass correlations for the following five categories of child social adversity are larger in identical than fraternal twins: bullying, sexual abuse, physical maltreatment, emotional neglect and abuse, and general trauma. Eleven relevant studies that encompassed 9119 twin pairs provided 24 comparisons of intraclass correlations, which we grouped into the five social exposure categories. Fisher’s *z*-test revealed significantly higher correlations in identical than fraternal pairs for each exposure category (*z* ≥ 3.53, *p* < 0.001). The difference remained consistent across gender, study site (country), sample size, whether psychometric instruments were used, whether interviewing was proximate or distant to the exposures, and whether informants were twins or third persons. Combined with other evidence that the differential intraclass correlation for child social adversity cannot be explained by evocative gene–environment covariation, our results indicate that the CTM does not provide any valid indication of genomic effects in schizophrenia.

## Introduction

A central impetus for contemporary molecular genetics searches of schizophrenia are estimates from behavioral genetics studies that the disorder is about 80% heritable (1). No comparable identification, not to say consistent replication, of causal genetic variants, however, has resulted. In a May 3rd, 2013 official press release, the leader of the DSM-5 Task Force acknowledged that, despite four decades of concerted effort, researchers have failed to identify any genetic variants that cause severe psychopathology. In 2014, the Schizophrenia Working Group of the Psychiatric Genomics Consortium published results from the largest genetic study of schizophrenia to date, reporting that 108 statistically significant single nucleotide polymorphisms (SNPs) together could account for only 3.4% of variation on the liability scale (2). Given the long history of failures to replicate initial findings in molecular genetics searches, it is premature to conclude that even this low level of explained variance does not represent false positives. Most importantly, the absence of direct genetic confirmation of anything close to the high heritability estimates from twin studies has led to the position that there is a missing heritability problem for schizophrenia as well as for mental disorders in general (3, 4).

A leading explanation for missing heritability is that a large number of genetic variants of small effect underlie various traits and disorders (4, 5). This hypothesis was investigated with genome-wide complex trait analysis (GCTA), which estimates the proportion of phenotypic variance explained by common SNPs (6). GCTA studies have reported substantial heritability estimates for a variety of traits, including psychosis (7), but also a lack of replication across studies (8). A problem with the GCTA method, however, is that its heritability estimates may be upwardly biased due to population substructures (stratification) (9). This may occur if genetic similarity between individuals in subgroups that differ in their mean levels on a trait correlates with environmental similarity (10–12). Since the informational value of GCTA studies currently is unclear, behavioral genetics studies still are central to the view of strong genetic effects in schizophrenia and other types of severe psychopathology.

Among the set of behavioral genetics methods that underlie the view of schizophrenia as highly heritable, the most frequently used and cited is the classical twin method (CTM). Another often used method, family studies, is generally considered as inadequate to differentiate between genetic and environmental influences. A stronger method would be to compare identical twins reared apart, which has the potential to disentangle genetic from environmental influences, but this design has not been applied to schizophrenia or any other psychotic disorders, with only single case studies of identical twins being published. A handful of other types of adoption designs have been used and are cited as having definitively established schizophrenia as a genetic disorder [e.g., Ref. (13–15)]. However, these studies contain a number of methodological issues and biases that question their potential to separate between genetic and environmental influences. This includes the tendency of adoption agencies and others to “selectively place” children into adoptive homes corresponding to the socioeconomic status and perceived genetic background of their biological parents, non-blinded diagnoses, the questionable use of broader “spectrum disorder” diagnoses, inconsistencies in the way adoptee, and relative group diagnoses were counted and assessed, the failure to find statistically significant results in some cases, investigator bias in favor of genetic interpretations, and the failure to study adoptees’ rearing environments [e.g., Ref. (16–19)]. On this basis, the CTM remains central to the view that schizophrenia is a highly heritable disorder.

The (classical) twin method compares the resemblance of reared-together identical (monozygotic, MZ) and same-sex fraternal twins (dizygotic, DZ) on a trait. Because identical twins share all their segregating genes, whereas fraternal twins share only half on average, twin researchers attribute the greater trait resemblance of identical twins to genetic factors. As for the more recent GCTA method, covariance will be a problem also for the CTM if identical twins are exposed to more similar environments than fraternal twins (20). Of particular concern, therefore, is that the CTM is based on the *assumption* that identical and fraternal twin pairs are not exposed to differentially correlated environments, the equal-environment assumption (EEA). According to the EEA, the environments of identical and same-sex fraternal twin pairs are so similar that the observed variance in behavioral phenotypes must derive overwhelmingly from genetic variance.

### The equal-environment assumption for schizophrenia

The CTM was designed to disentangle genetic from environmental effects, with the goal being to estimate the role of trait variance explainable by genetic variation (heritability). Although heritability is commonly understood as meaning that genetic factors play a role in causing a trait or condition, strictly speaking the term refers only to the percentage of trait variation in a population statistically associated with genetic factors, which is what we refer to here. Some critics have questioned the validity of the use of heritability estimates in the behavioral sciences (17, 18, 21, 22). By evoking the EEA and drawing on the higher genetic similarity in identical than fraternal twins, twin researchers typically have calculated heritability estimates by doubling the correlation or concordance rate difference between identical and fraternal pairs (23). Over the past few decades, more complex biometrical analyses have been used to partition genetic (A), “shared environment” (C), and “unshared environment” (E) contributions to trait variation in a population [the “ACE Model,” Ref. (24)]. In the absence of environmental measures, twin researchers use maximum likelihood statistics to estimate genetic and environmental effects. However, regardless of the apparent complexity of the models, path diagrams, twin family designs, and so on, all are based on the validity of the EEA. If the EEA is violated then the ACE model – which assumes clear separation of variance A from variances C and E – becomes infeasible.

During the first 45 years of the twin method (roughly 1924–1969), the assumption that identical and fraternal pairs experience roughly equal environments became infeasible (22, 25). That is, the evidence showed that identical pairs are treated more alike and are socialized to be more alike than are fraternal pairs (17). Twin researchers Scarr and Carter-Saltzman (26) (p. 528) concluded, “the evidence of greater environmental similarity for MZ than DZ twins is overwhelming.” According to twin researcher John Loehlin, in his 1976 study, he “found, as nearly everybody else has found who has investigated the point, that identical twins are indeed treated more alike – they are dressed alike more often, are more often together at school, play together more, and so forth” (27) (p. 72). Others have pointed to identical twins’ greater psychological closeness, identity confusion, and “ego fusion” when compared with fraternal pairs (28).

In response to the overwhelming evidence that identical twins experience more similar environments than fraternals, twin researchers changed the traditional EEA by adding the condition that what matters is whether identical pairs experience more similar environments that are relevant to the trait in question (29). As formulated by Kendler, Neale, Kessler, Heath, and Eaves (30) (p. 21):

The traditional twin method, as well as more recent biometrical models for twin analysis, are predicated on the EEA – that monozygotic (MZ) and dizygotic (DZ) twins are equally correlated for their exposure to environmental influences that are of etiologic relevance to the trait under study.

In light of this trait-relevant specification, for the EEA to be valid for schizophrenia, identical twins must not be more similar on etiologically relevant environmental exposures. Among the range of environmental variables that appear to be etiologically relevant for schizophrenia are prenatal stress and infections, inflammation, and adolescent substance abuse (31–33). Most crucially, however, an array of studies has established that child social adversity is particularly trait-relevant to the development of schizophrenia and psychotic symptoms (34, 35). Central among these adversities are emotional abuse and neglect, sexual and physical abuse, bullying, and poverty (34–38). These child adversities have been associated with schizophrenia and/or schizophreniform psychotic symptoms in both retrospective and prospective studies, including prospective studies that used twin designs (39). Population based studies consistently have reported dose–effect associations between the number of childhood adversities experienced and the prevalence of schizophreniform psychosis and psychotic symptoms (40, 41).

When the EEA has been empirically tested in twin studies of schizophrenia or any other types of psychosis, researchers have failed to assess whether identical and fraternal pairs experience different levels of exposure to the child social adversities listed above or any other type of social adversity. To the best of our knowledge, only one test of the EEA in psychosis has been reported in the literature. This is an unpublished study by Kendler and Robinette that was referred to in a 1983 paper by Kendler [see Table 1 in Ref. (42)], which focused on the degree of twin physical similarity. Twin physical similarity has never been associated with schizophrenia in any study, which makes Kendler and Robinette’s results of indirect value at best, an issue we return to in our discussion.

The validity of the (trait-relevant) EEA in schizophrenia can be partially assessed by determining whether identical and fraternal twin pairs drawn from the general population of twins experience similar or differing levels of adverse social exposures during upbringing. That is, in the general bulk of published twin studies, do identical pairs tend to be more similarly exposed than fraternal pairs to social adversity during childhood? If this is the case, an unequal environment would be likely to contribute to the higher concordance rates for schizophrenia in identical as compared to fraternal twins, undermining the validity of the CTM. On this basis, we reviewed the empirical literature of twin studies to provide the first (to our knowledge) meta-analysis of whether identical and fraternal twin pairs are equally correlated on child social adversities. We reviewed twin studies where the authors reported intraclass correlations for identical and fraternal pairs for the following child adversities, all being robustly associated with schizophrenia and psychotic symptoms in the general scientific literature: (1) sexual abuse, (2) physical abuse/physical maltreatment, (3) emotional abuse and neglect, (4) bullying, and (5) traumatic life events.

In finding that schizophrenia-relevant social adversities are systematically *more similar* in identical than fraternal pairs in general populations of twins, we discuss two main counter arguments used by twin researchers when the EEA is violated. First, that tests of the EEA for *other* traits than schizophrenia (and other traits than psychosis in general) by the use of social exposures that are *not* etiologically relevant for this disorder support the continued use of the assumption. Second, that increased trait-relevant exposures in identical as compared to fraternal twin pairs reflect that identical twins have created more similar environments for themselves because they are more similar genetically.

## Method

We searched PubMED and PsycINFO for peer reviewed articles published in English between January 1 1990 and November 1 2014 using combinations of the following search words: “twin*,” “bullying,” “sexual,” “physical,” “maltreatment,” “abuse,” “punishment,” “emotion*,” “neglect,” “stress*,” “life events,” “adverse,” “trauma.” Subsequent searches for studies were performed based on the reference lists of the identified primary studies and overview papers.

We used the following inclusion criteria for original articles and studies: (1) measures were taken of social adversities that could be classified as either of sexual abuse, physical abuse or maltreatment, emotional abuse or neglect, bullying, and traumatic life events; (2) the social adversities occurred during childhood or adolescence [due to a low number of studies, however, this criterion was relaxed to mean that childhood and adolescence (before age 18) should be included in the measured exposure period]; (3) intraclass correlations were presented for social adversities for both identical and fraternal twins; and (4) the number of identical and fraternal twin pairs was stated, which was necessary for our statistical analysis.

The initial search yielded more than 2,000 hits. Only 22 articles reported upon intraclass correlations for any type of adverse social exposures in identical and fraternal pairs. Among these, 14 articles met our inclusion criteria, reporting from 11 unique studies (39, 43–55). Three articles reported data on partly overlapping types of adversities from the same study (39, 43, 44), and two other articles from another study reported data on the same exposure types but on different sample sizes (52, 55). As a result, 12 articles with unique data from 11 studies remained.

### Quality criteria – external validity

In none of the identified studies was the primary aim to investigate and compare intraclass correlations for social adversities in identical and fraternal twins. Neither did any of the studies aim at testing the EEA. Instead, intraclass correlations for social adversities were presented in the context of modeling of genetic (and environmental) effects on various mental and somatic disorders, investigating gene–environment interactions, and assessing genetic influences on environmental exposures. An evaluation of the overall quality of the studies therefore was not relevant to our purpose. Instead, we focused our quality assessment on the external validity and generalizability of the overall results of differences in the intraclass correlations. We examined whether the general findings from the 11 studies were generalizable across the following six methodological criteria or variables: (1) social adversity as measured using validated instruments or not, (2) informants of social adversity being the twins themselves or third persons (parents, teachers), (3) sample size, comparing the subset of studies with sample sizes above and below the grand median, (4) interviews for social adversities performed during childhood/adolescence (proximate to their occurrence, participant age <19 years) or in adulthood (distant from their occurrence, participant age >22 years), (5) gender (male, female), and (6) site/country where the study was executed (US/Canada versus Europe/Australia).

### Statistical analysis

The hypothesis that intraclass correlations for five categories of social adversities are not higher in identical than fraternal twins was tested using Fisher’s (56) method to compare the size of two correlations. The method first transforms each correlation (*r* to *r*′) by the formula

$$\text{r}\prime =\left(0.\text{5}\right)\text{ lo}{\text{g}}_{\text{e}}\left(\text{1}+\text{r}/\text{1}-\text{r}\right)$$

Next, the *z*-statistic is computed by entering the transformed values for the correlations to be compared $\left(r_{\text{identical}}^{\prime }\text{ and }r_{\text{fraternal}}^{\prime }\right)$ into the formula

$$z=\left(r_{\text{identical}}^{\prime }-r_{\text{fraternal}}^{\prime }\right)\;/(\sqrt{}\left[\left(\text{1}/n_{\text{identical}}-\text{3}\right)\;+\;\left(\text{1}/n_{\text{fraternal}}-\text{3}\right)\right]$$

Both of these steps and the subsequent determination of the probability for *z* under the null hypothesis (calculated from the cumulative normal distribution), can be executed with freely available software (e.g., http://vassarstats.net/rdiff.html).

Fisher *z*-tests were first performed for each individual comparison of intraclass correlations for social adversities that were presented in the 11 identified studies. Second, the same test was performed on our five main constructs for social adversities. In the latter analyses, we used weighted correlation values for each construct. To obtain weighted intraclass correlations for each twin type, we first multiplied each correlation coefficient with the number of twin pairs used in that correlation (e.g., *r* for identical twins multiplied by number of identical twin pairs). Next, we summed up the resulting product values over all comparisons involved for that construct (separately for each twin type), and finally divided the sum of the product values with the sum of the number of participants in all of the comparisons. To obtain *z*-scores and *p*-values, we entered into the calculation the total number of *unique* twin pairs that were involved in the included set of comparisons for that construct, i.e., we summed *n* across (involved) studies and not across individual correlations.

To assess the external validity/generalizability of the findings across the six methodological variables noted above, we first dichotomized each variable to obtain 12 subgroups for the overall set of pairs of intraclass correlations. These categorizations of the intraclass correlations were done independent of type of adversity, with all adversity types collapsed. Fisher *z*-test was performed for each of the 12 involved subgroups, using weighted correlation coefficients as described above.

## Results

The 12 articles derived from the 11 unique studies in our primary review encompassed a total of 24 comparisons of intraclass correlations for social adversities in identical pairs and fraternal pairs. The comparisons included a total of 9119 twin pairs (5187 identical pairs and 3932 fraternal pairs, see Table 1). Sample size ranged from 150 to 1502 twin pairs (mean = 829, SD = 534).

**Table 1:** **Characteristics of 11 included studies**.

Study	*N* twin pairs – identical, fraternal	Social adversity constructs	Age of exposure (years)	Age of interview (years)	Informants
E-Risk study Ball et al. (39), Jaffee et al. (43, 44), UK	625, 491	Bullying victimization, physical maltreatment, corporal punishment	<10	5–10	Parents, teachers
Shakoor et al. (45), UK	709, 629	Bullying victimization	<12	12	Twins
Bornovalova et al. (46), US	896, 486	Sexual, physical, and emotional abuse	<18	24	Twins
Dinwiddie et al. (47), Australia	923, 525	Forced into sexual activity	<18	Adults, mean ~45	Twins
Jay Schulz-Heik et al. (48), US	271, 397	Physical maltreatment and neglect by parents or other adult caregivers	<12	Young adults, mean 22.5	Twins
Lau and Eley (49), UK	267, 328	Negative life events	<12–19	12–19	Twins
Rooks et al. (50), US	146, 95	Accumulated trauma: physical, sexual, and emotional abuse and general trauma	<18	Middle aged, mean 55	Twins
Silberg et al. (51), US	185, 88	Stressful life events	Past year (<8–16)	8–16	Mother
Stein et al. (52), Canada	222, 184	Assaultive and non-assaultive trauma	Sum of three age periods: 0–6, 7–16, 17–present	Adults, mean ~33–34	Twins
Vinkhuyzen et al. (53), Netherlands	83, 67	Being bullied at primary and secondary school, negative life events	<18	Adults, mean 47	Twins
Young-Wolff et al. (54), US	860, 642	Composite measure of child maltreatment	<15	Adults, mean 35	Twins

Twenty-three of the 24 intraclass correlations (96%) were nominally higher in identical than fraternal pairs. The hypothesis that the intraclass correlation was not higher in identical than fraternal twins was rejected at the *p* = 0.05 level (*z* > 1.282) for 16 of the 24 comparisons (67%) (Table 2). The one correlation that was nominally higher in fraternal pairs than identical pairs was not statistically significant (*r* = 0.56 versus *r* = 0.55).

**Table 2:** **Intraclass correlations for schizophrenia-relevant adverse social experiences during childhood or adolescence**.

Study	Operational characteristics of variables	Intraclass correlations	Fisher’s *Z*, *p*
**BULLYING VICTIMIZATION**			
Ball et al. (39)	A question about whether either twin had been bullied by another child since they were age 5	Boys: *r*_ident_ = 0.77; *r*_frat_ = 0.41 Girls: *r*_ident_ = 0.65; *r*_frat_ = 0.36	9.67, <0.0001 6.59, <0.0001
Shakoor et al. (45)	Total scores on the 16-item Multidimensional Peer Victimization Scale	*r*_ident_ = 0.62; *r*_frat_ = 0.42	5.05, <0.0001
Vinkhuyzen et al. (53)	A question about whether they had been bullied at primary and/or secondary school	Primary school: *r*_ident_ = 0.74; *r*_frat_ = 0.41 Secondary school: *r*_ident_ = 0.52; *r*_frat_ = 0.23	3.07, 0.001 2.04, 0.021
**SEXUAL ABUSE**			
Bornovalova et al. (46)	Confirmatory answers on questions of sexual abuse on either the Trauma Assessment for Adults (three items) or the Childhood Experiences Questionnaire (CEQ) (seven items). Answers on scales were combined.	*r*_ident_ = 0.60; *r*_frat_ = 0.58	0.54, ns^a^
Dinwiddie et al. (47)	One item: “Before age 18, were you ever forced into sexual activity, including intercourse?”	*r*_ident_ = 0.68; *r*_frat_ = 0.52	4.61, <0.0001
**PHYSICAL MALTREATMENT**			
Bornovalova et al. (46)	Four items from the CEQ, with two items for physical abuse (hit with a weapon; kicking, biting or burning) and two items on harsh discipline (hit with a hand or fist more than once; hit with an object more than once)	*r*_ident_ = 0.61; *r*_frat_ = 0.56	1.35, 0.089
Jaffee et al. (43, 44)	Questions about physical maltreatment and corporal punishment based on the clinical interview protocol from the Multi-Site Child Development project. Standardized probe questions about the child being physically harmed/hurt – either related to or independent of disciplinary practices, followed by details about the incidents	Maltreatment: *r*_ident_ = 0.77; *r*_frat_ = 0.71 Punishment: *r*_ident_ = 0.90; *r*_frat_ = 0.79	2.20, 0.014 6.63, <0.0001
Jay Schulz-Heik et al. (48)	One question:”How often had your parents or other adult care-givers slapped, hit, or kicked you?”	Males: *r*_ident_ = 0.33; *r*_frat_ = 0.03 Females: *r*_ident_ = 0.55; *r*_frat_ = 0.56	3.95, <0.001 −0.18, ns
**EMOTIONAL ABUSE AND NEGLECT**			
Jay Schulz-Heik et al. (48)	Two questions on how often parents or other adult care-givers had (a) left the twin alone when an adult should have been with them and (b) not taken care of the twin’s basic needs, such as keeping them clean or providing food or clothing	Males: *r*_ident_ = 0.31; *r*_frat_ = 0.28 Females: *r*_ident_ = 0.41; *r*_frat_ = 0.20	0.42, ns 2.84, 0.0016
Bornovalova et al. (46)	Six items on the CEQ on emotional abuse, including insult, ridicule, humiliation, and tormenting with scary things	*r*_ident_ = 0.53; *r*_frat_ = 0.36	3.78, 0.0001
**COMBINED PHYSICAL AND SEXUAL ABUSE AND NEGLECT**			
Young-Wolff et al. (54)	Combined score based on three questions: “Have you ever been physically abused,” “Have you ever been sexually abused or molested,” and “Were you ever seriously neglected as a child”	*r*_ident_ = 0.64; *r*_frat_ = 0.57	2.12, 0.017
**STRESSFUL/NEGATIVE LIFE EVENTS/TRAUMA IN GENERAL**			
Silberg et al. (51)	A subset of negative life events drawn from a list of 39 past year life events including quarreling with parents, breaking up with someone, losing a friend, and failing a grade	First time point: *r*_ident_ = 0.91; *r*_frat_ = 0.75 1.5 years later: *r*_ident_ = 0.89; *r*_frat_ = 0.52	4.22, <0.0001 6.44, <0.0001
Stein et al. (52)	Eight items from the Traumatic Event Questionnaire to assess Assaultive trauma (robbery, being held captive, being beaten up, sexual assault, and other potentially life threatening events) and non-assaultive trauma (sudden family death, motor vehicle accident, fire, tornado, flood, earthquake	Assaultive trauma: *r*_ident_ = 0.43; *r*_frat_ = 0.28 Non-assaultive trauma: *r*_ident_ = 0.39; *r*_frat_ = 0.25	1.71, 0.044 1.56, 0.059
Lau and Eley (49)	Sum of 12 negative life events derived from the Life Events Scale for Adolescents and that could be related to each individual twin (e.g., break-up with boy/girlfriend)	Males: *r*_ident_ = 0.57; *r*_frat_ = 0.42 Females: *r*_ident_ = 0.50; *r*_frat_ = 0.41	2.41, 0.008 1.37, 0.085
Vinkhuyzen et al. (53)	Sum of items about negative life events from the List of Threatening Experiences, including severe illness, violent assault, robbery, severe trouble with friends, and sexual abuse	*r*_ident_ = 0.50; *r*_frat_ = 0.44	0.46, ns
Rooks et al. (50)	Total scores on the Early Trauma Inventory, including physical, sexual, and emotional abuse and general trauma. Scores were dichotomized as yes – no based on mean scores for a group of individuals without psychopathology	*r*_ident_ = 0.54; *r*_frat_ = 0.47	0.70, ns

*^a^ns = not significant*.

The 24 pairs of intraclass correlations were sorted into the five general categories for social adversities that we focused on in the review (sexual abuse, physical maltreatment, bullying, emotional abuse or neglect, and general trauma), following their operational characteristics as described in Table 2, second column. For each of the five general constructs, the hypothesis that the intraclass correlation was not higher in identical than fraternal twins was rejected at the *p* < 0.001 level of significance, with *z*-values ranging from 3.53 to 10.13 (Table 3).

**Table 3:** **Comparison of weighted intraclass correlations for categories of social adversities**.

Main construct	Unique twin pairs included (n) – identical, fraternal	Comparisons in analysis (*n*)	Weighted intraclass correlations	Fisher’s *Z*, *p*
Bullying	1417, 1187	4	*r*_ident_ = 0.67; *r*_frat_ = 0.39	10.13, <0.001
Sexual abuse	1749, 1011	2	*r*_ident_ = 0.64; *r*_frat_ = 0.55	3.53, <0.001
Physical maltreatment	1792, 1374	5	*r*_ident_ = 0.68; *r*_frat_ = 0.55	5.87, <0.001
Emotional abuse and neglect	1167, 883	3	*r*_ident_ = 0.47; *r*_frat_ = 0.29	4.73, <0.001
Stressful/traumatic life events	903, 762	8	*r*_ident_ = 0.58; *r*_frat_ = 0.41	4.60, <0.001

The significance of the difference in intraclass correlations between identical pairs and fraternal pairs did not depend on any of the six parameters/criteria that we analyzed. For all 12 comparisons of intraclass correlations involved for these six parameters, identical pairs were more similar on social adversities than fraternal pairs at the *p* < 0.001 level of significance, with *z*-scores ranging from 3.76 to 10.75 (Table 4).

**Table 4:** **Influence of six parameters on the difference in intraclass correlations**.

Parameter	Condition	Number of unique twin pairs – identical, fraternal	Number of comparisons	Weighted intraclass correlations (identical, fraternal)	Fisher’s *Z*, *p*
Use of psychometric instrument for social adversity	Yes	2951, 2280	14	*r*_ident_ = 0.62; *r*_frat_ = 0.51	5.82, <0.001
	No	2864, 2143	10	*r*_ident_ = 0.63; *r*_frat_ = 0.40	9.79, <0.001
Informants of social adversity	Twins	5005, 3844	20	*r*_ident_ = 0.60; *r*_frat_ = 0.46	9.15, <0.001
	Third person	810, 579	4	*r*_ident_ = 0.75; *r*_frat_ = 0.42	9.63, <0.001
Sample size	Below median	910, 831	12	*r*_ident_ = 0.51; *r*_frat_ = 0.31	5.04, <0.001
	Above median	4460, 3101	12	*r*_ident_ = 0.66; *r*_frat_ = 0.51	9.84, <0.001
Age of interviewing	Age ≤19 years	1786, 1536	9	*r*_ident_ = 0.73; *r*_frat_ = 0.51	10.51, <0.001
	Age >22 years	3404, 2396	15	*r*_ident_ = 0.56; *r*_frat_ = 0.41	7.39, <0.001
Gender	Male	502, 498	4	*r*_ident_ = 0.56; *r*_frat_ = 0.31	4.92, <0.001
	Female	660, 544	4	*r*_ident_ = 0.55; *r*_frat_ = 0.38	3.76, <0.001
Site of study/country	US or Canada	2583, 1892	13	*r*_ident_ = 0.56; *r*_frat_ = 0.41	6.51, <0.001
	Europe, Australia	2607, 2040	11	*r*_ident_ = 0.70; *r*_frat_ = 0.50	10.75, <0.001

## Discussion

Data from 9119 twin pairs included in 24 comparisons of intraclass correlations in 11 studies rejected the assumption that identical as compared to fraternal twins are equally correlated on child social adversities that are etiologically relevant to schizophrenia. Identical twin pairs were more similar than fraternal pairs on bullying, sexual abuse, physical maltreatment, emotional abuse and neglect, and general negative life events or trauma. The findings were generalizable across a range of potentially moderating parameters and imply that the EEA is invalid for schizophrenia and other disorders where child adversity is a trait-relevant exposure.

Below, we first discuss the common objection from twin researchers that it is not necessary to test the EEA with relevant social exposures since the use of trait-irrelevant exposures has shown that the EEA is valid for all mental disorders as well as other psychological and behavioral traits. Second, we discuss twin researchers’ claim that it does not matter if the EEA is violated because the more similar child adversities in identical than fraternal twin pairs reflect the more similar genetically based impact of identical twins upon their environment, and thus can be treated as a genetic effect.

### The relevance of EEA tests based on etiologically non-relevant social exposures

When twin researchers have tested the EEA for mental disorders, they have measured environmental aspects that have not been linked to the disorders, most typically whether parents correctly identified the zygosity of the twins, the degree of twin similarity in physical appearance, and whether identical twins are dressed more alike (57, 58). The use of these types of measures has been predicated on the assumption that the measures correlate with similarity in treatment of the twins by parents and others. When such measures have not been found to be substantially associated with trait correlations among twins, researchers have concluded that the EEA is valid.

Regarding schizophrenia, the relevant question would be whether variables such as twin physical similarity and being dressed alike correlate with child social adversities. Consider the example of a group of identical twins: it is known that these twins on average are dressed more similarly and look more similar than fraternal twins. The question is whether variations in similarity of appearance and ways of being dressed *within* a group of identical twins (or fraternal twins) are associated with the presence versus absence of people who treat the twins adversely. This is an empirical question, which does not seem to have been addressed by twin researchers. We suspect that there will be no such correlations, hence that these “EEA tests” are irrelevant.

Twin researchers claim that studies where parents or twins themselves have misclassified twin zygosity show that the EEA is valid. The assumption in these studies is that if it is the parents’ treatment of the twins and not the twins’ genes that is etiologically important, then fraternal twins that are misclassified as identical should correlate higher for mental disorders than twins correctly classified as fraternal, whereas identical twins misclassified as fraternal should correlate lower than correctly classified identical twins. When twin researchers have not found this pattern of correlations for mental disorders, they have concluded that it is not the parents’ treatment but the twins’ genes that are important. However, the misclassification test is based on the assumption that it is the parents’ or twins’ beliefs about twin zygosity that underlie how similarly they (or other people) expose the twins for adverse treatments. Again, this is a hypothesis that twin researchers have not tested empirically, making them unable to reject the alternative hypothesis that erroneous beliefs about twin zygosity is not associated with the similarity of adverse treatments of the twins.

### The role of evocative gene–environment covariation

In situations where the EEA is violated, twin researchers have put forward the hypothesis that, due to their more similar genomes, identical twins elicit more similar environments for themselves than fraternal twins, termed evocative correlation (59). When (trait-relevant) exposures are found to correlate more strongly in identical than fraternal twins, so that the EEA is violated, it can be countered that the increased similarity in identical pairs really is a genetic effect (18). In the words of twin researchers Bouchard, Lykken, McGue, Segal, and Tellegen (60) (p. 227–228); “Twins tend to elicit, select, seek out, or create very similar effective environments and, to that extent, the impact of these experiences is counted as genetic influence.”

Genetically influenced individual characteristics, such as physical appearance and temperament, contribute to the treatment received from other people. However, for the evocative correlation hypothesis to be a valid defense of the twin method, a prerequisite is that the increased correlation in child trait-relevant exposures in identical as compared to fraternal twins predominantly results from more similar evocative influences in the former. That is, child victims themselves must be considered the primary causal agents when exposed to adverse treatments such as physical and sexual abuse and bullying.

The role of evocative influences on adverse social exposures in childhood and adolescence has been estimated in an array of twin studies and other types of behavioral genetics designs. In a 2007 meta-analysis of 55 behavioral genetic studies, Kendler and Baker (61) calculated the degree of genetically based evocative influence on a range of social exposures that they held to be relevant for psychiatric disorders. They estimated a weighted heritability for social exposure to 27%. Thus, 73% of the variance remained unexplained by genetics, which indicates that the evocative influence argument is an insufficient defense of the EEA. Even the 27% figure that Kendler and Baker attributed to genetics is of questionable relevance: when behavioral genetics researchers have assessed for the role of evocative influences on social exposures the same logic and statistical estimation techniques typically used to estimate heritability for psychological and behavioral traits have been applied. That is, in twin studies they have evoked the EEA as a rationale for ascribing twice the difference in intraclass correlations for *social exposures* between identical and fraternal twins to evocative genetic influence. The validity of the EEA for psychiatric disorders naturally cannot logically be investigated by studies that *employ* the EEA at another level of analysis – for child social exposures.

Findings that much of the variance in child social adversities cannot be explained by evocative factors have been reported from twin studies that used other types of estimation techniques. Two examples are particularly pertinent since they focused on psychotic symptoms. First, Arsenault et al. (62) reported that 12-year-old twins who had been exposed to both bullying and maltreatment had a 5.7 times higher risk for psychosis symptoms than twins exposed to no adversity. In a second step, they “controlled” for genetic influence by treating the genetic risk as higher if an identical as compared to fraternal co-twin experienced psychotic symptoms, a strategy that reflects their use of the EEA. In spite of this “control” for genetic influences twins who experienced both types of adversities still were 3.8 times more likely to have psychotic symptoms than twins with no adverse experience. Second, in a study that focused on pairs of identical twins, Alemany et al. (63) reasoned that if *differences* between identical twins in social adversity are associated with their differences in psychosis symptoms, this difference-by-difference association cannot be accounted for by genetic influence. Alemany et al. (63) indeed found that differences between their identical twins in child adversity (abuse and neglect) were significantly associated with differences in their scores for both positive and negative psychotic experiences. These findings strongly suggest that the evocative influence argument does not function as a defense of the EEA for psychotic disorders.

Other types of studies have documented that adverse treatment of children is robustly associated with the manifestations of psychopathology in the perpetrator, deviant personality, substance abuse, harsh disciplinary strategies, and marital problems (64). Likewise, insecure or dismissing parental attachment styles, which are associated with the development of a range of mental disorders, do not primarily reflect child influence (65). Also, studies of parents who are twins have found that child rearing is significantly associated with parental characteristics (66). Along the same lines, although some children may be more prone to bullying by peers than others, bullying is associated with characteristics of the perpetrators and their psychosocial circumstances (67).

That stress exposure can be discarded if the individual contributed to its occurrence, by itself is a dubious notion; stress has real effects whatever its causes. The findings noted above that child adversities contribute independently to psychotic symptoms (62, 63) are corroborated by a long array of other research in humans and animals that stress impacts upon neurophysiological and behavioral development. For example, in humans, severe child adversity alters brain development in ways that are congruent with the changes seen in schizophrenia, including altered structure and functioning of the HPA stress axis, hippocampus, dopaminergic striatum, and prefrontal cortex (68–70). Cross-fostering studies in animals have shown that early adverse treatment rather than genetic variation underlie the development of deviant behavior and associated brain abnormalities, lasting into adult life (71). Consistently, sensitivity to stress is a central capacity across phylogenesis and that includes remodulation of epigenetic states, altered gene transcription rates, and perhaps also structural changes in DNA (mutagenesis) under stress exposure (72–74). The robust effects of stress imply that the important question is whether stress occurs and not what its causes are, which makes the evocative influence argument largely irrelevant. To use an analogy, it would not matter whether or not a deadly assault followed an individual’s deliberate choice of going into a dark alley; the effects of the assault would still be real.

### Implications

One implication from our findings is that future twin studies of schizophrenia at a minimum should include measures of the five categories of social adversities that we have focused on. However, it is unlikely that this will adequately capture relevant social exposures. All five categories vary along several dimensions in addition to their prevalence, including intensity, age of occurrence, duration, repetition, and number of perpetrators. Other exposure types also needs to be included for a full exploration and capture of social influences, such as prenatal exposures, substance abuse, and subtle family characteristics such as general relationship problems with parents, punitive discipline, and over controlling and cold parenting – all being reported to be more similar in identical pairs than fraternal pairs (49, 75–77). Moreover, the social exposures that are trait relevant to schizophrenia operate in complex interactions with other factors. Examples are whether children blamed themselves for the adversities, their coping resources and ability to solve the impact of the trauma, and resilience factors such as good social networks and safe attachment figures (78). Even if such issues were included in future twin studies, other problems would remain, such as interaction effects between environments and genetic contributions to normal variations in psychological traits (for example temperament), which should not be treated as main genetic effects. Most importantly, since the extensive set of relevant social variables and their interaction have been close to totally ignored in all classical twin studies of schizophrenia to date, the interpretation of genetic effects from these studies has questionable merit.

The danger of premature attribution to genetic causes of schizophrenia is that of undue closure around important questions. The true nature of the condition, and its etiology, are still largely unknown and must remain open questions, free, as far as possible, from subjective presuppositions, especially theoretically biased ones. Our study will hopefully contribute to that spirit of openness and help preclude misinterpretation of data based on flawed methodology.

## Conclusion

When identical twins are more similar on the most etiologically relevant social exposures for schizophrenia, it is irrational to continue with the notion that the EEA is valid for this disorder. Since the EEA is “crucial to everything that follows from twin research,” (79) (p. 155) its systematic violation for core social exposures implies that classical twin studies cannot provide any valid indication of genomic effects on schizophrenia and should be abandoned.

## Conflict of Interest Statement

The authors declare that the research was conducted in the absence of any commercial or financial relationships that could be construed as a potential conflict of interest.

## References

[1] Gershon ES Alliey-Rodriguez N Liu C. After GWAS: searching for genetic risk for schizophrenia and bipolar disorder. Am J Psychiatry (2011) 168(3):253–6. 10.1176/appi.ajp.2010.10091340 21285144 PMC6760656

[2] Schizophrenia Working Group of the Psychiatric Genomics Consortium. Biological insights from 108 schizophrenia-associated genetic loci. Nature (2014) 511(7510):421–7. 10.1038/nature13595 25056061 PMC4112379

[3] Maher B Personal genomes: the case of the missing heritability. Nature (2008) 456(7218):18–21 10.1038/456018a 18987709

[4] Eichler EE Flint J Gibson G Kong A Leal SM Moore JH Missing heritability and strategies for finding the underlying causes of complex disease. Nat Rev Genet (2010) 11(6):446–50. 10.1038/nrg2809 20479774 PMC2942068

[5] Manolio TA Collins FS Cox NJ Goldstein DB Hindorff LA Hunter DJ Finding the missing heritability of complex diseases. Nature (2009) 461(7265):747–53 10.1038/nature08494 19812666 PMC2831613

[6] Yang J Benyamin B McEvoy BP Gordon S Henders AK Nyholt DR Common SNPs explain a large proportion of the heritability for human height. Nat Genet (2010) 42(7):565–9 10.1038/ng.608 20562875 PMC3232052

[7] Lee SH DeCandia TR Ripke S Yang J Sullivan PF Goddard ME Estimating the proportion of variation in susceptibility to schizophrenia captured by common SNPs. Nat Genet (2012) 44(3):247–50. 10.1038/ng.1108 22344220 PMC3327879

[8] Trzaskowski M Dale PS Plomin R. No genetic influence for childhood behavior problems from DNA analysis. J Am Acad Child Adolesc Psychiatry (2013) 52(10):1048–56 e3. 10.1016/j.jaac.2013.07.016 24074471 PMC3914760

[9] Charney E Still Chasing Ghosts: A New Genetic Methodology Will Not Find the “Missing Heritability”: The Bioscience Resource Project (2013). Available from: http://www.independentsciencenews.org/health/still-chasing-ghosts-a-new-genetic-methodology-will-not-find-the-missing-heritability/

[10] Browning SR Browning BL. Identity-by-descent-based heritability analysis in the Northern Finland birth cohort. Hum Genet (2013) 132(2):129–38. 10.1007/s00439-012-1230-y 23052944 PMC3543768

[11] Browning SR Browning BL Population structure can inflate SNP-based heritability estimates. Am J Hum Genet (2011) 89(1):191–3 10.1016/j.ajhg.2011.05.025 21763486 PMC3135810

[12] Janss L de Los Campos G Sheehan N Sorensen D. Inferences from genomic models in stratified populations. Genetics (2012) 192(2):693–704. 10.1534/genetics.112.141143 22813891 PMC3454890

[13] Kety SS Rosenthal D Wender PH Schulsinger F The types and prevalence of mental illness in the biological and adoptive families of adopted schizophrenics. In: Rosenthal D Kety SS, editors. The Transmission of Schizophrenia. New York, NY: Pergamon Press (1968). p. 345–62.

[14] Kety SS Wender PH Jacobsen B Ingraham LJ Jansson L Faber B Mental illness in the biological and adoptive relatives of schizophrenic adoptees. Replication of the Copenhagen study in the rest of Denmark. Arch Gen Psychiatry (1994) 51(6):442–55. 10.1001/archpsyc.1994.03950060006001 8192547

[15] Rosenthal D Wender PH Kety SS Welner J Schulsinger F The adopted-away offspring of schizophrenics. Am J Psychiatry (1971) 128(3):307–11 10.1176/ajp.128.3.307 5570995

[16] Jackson G Rethinking the Finnish adoption studies of schizophrenia: a challenge to genetic determinism. J Crit Psychol Couns Psychother (2003) 3:129–38.

[17] Joseph J The Gene Illusion: Genetic Research in Psychiatry and Psychology Under the Microscope. New York, NY: Algora (2004).

[18] Joseph J The “missing heritability” of psychiatric disorders: elusive genes or non-existent genes? Appl Dev Sci (2012) 16:65–83 10.1080/10888691.2012.667343

[19] Lewontin RC Rose S Kamin LJ Not in Our Genes: Biology, Ideology and Human Nature. New York, NY: Pantheon (1984).

[20] Kendler KS Gardner CO Jr. Twin studies of adult psychiatric and substance dependence disorders: are they biased by differences in the environmental experiences of monozygotic and dizygotic twins in childhood and adolescence? Psychol Med (1998) 28(3):625–33. 10.1017/S0033291798006643 9626718

[21] Lewontin RC Rose S Kamin LJ Not in Our Genes. New York, NY: Pantheon (1984).

[22] Joseph J The Trouble with Twin Studies: A Reassessment of Twin Research in the Social and Behavioral Sciences. New York, NY: Routledge (2015).

[23] Boomsma D Busjahn A Peltonen L Classical twin studies and beyond. Nat Rev Genet (2002) 3(11):872–82 10.1038/nrg932 12415317

[24] Medland SE Hatemi PK Political science, biometric theory, and twin studies: a methodological introduction. Political Anal (2009) 17:191–214 10.1093/pan/mpn016

[25] Joseph J The use of the classical twin method in the behavioral sciences: the fallacy continues. J Mind Behav (2013) 34:1–40.

[26] Scarr S Carter-Saltzman L. Twin method: defense of a critical assumption. Behav Genet (1979) 9(6):527–42. 10.1007/BF01067349 263638

[27] Loehlin JC Identical twins reared apart and other routes to the same direction. In: Nance W Allen G Parisi P, editors. Twin Research, Part A: Psychology and Methodology. New York, NY: Allan R. Liss (1978). p. 69–77. 568284

[28] Dalgard OS Kringlen E. A Norwegian twin study of criminality. Br J Criminol (1976) 16:213–32.12098908

[29] Gottesman II Shields J Contributions of twin studies to perspectives on schizophrenia. Prog Exp Pers Res (1966) 3:1–84. 5337692

[30] Kendler KS Neale MC Kessler RC Heath AC Eaves LJ. A test of the equal-environment assumption in twin studies of psychiatric illness. Behav Genet (1993) 23(1):21–7. 10.1007/BF01067551 8476388

[31] Burns JK. Pathways from *Cannabis* to psychosis: a review of the evidence. Front Psychiatry (2013) 4:128. 10.3389/fpsyt.2013.00128 24133460 PMC3796266

[32] Suvisaari J Mantere O. Inflammation theories in psychotic disorders: a critical review. Infect Disord Drug Targets (2013) 13(1):59–70. 10.2174/18715265112129990032 23713669

[33] Markham JA Koenig JI. Prenatal stress: role in psychotic and depressive diseases. Psychopharmacology (Berl) (2011) 214(1):89–106. 10.1007/s00213-010-2035-0 20949351 PMC3050113

[34] Varese F Smeets F Drukker M Lieverse R Lataster T Viechtbauer W Childhood adversities increase the risk of psychosis: a meta-analysis of patient-control, prospective- and cross-sectional cohort studies. Schizophr Bull (2012) 38(4):661–71. 10.1093/schbul/sbs050 22461484 PMC3406538

[35] Read J Bentall RP Fosse R. Time to abandon the bio-bio-bio model of psychosis: exploring the epigenetic and psychological mechanisms by which adverse life events lead to psychotic symptoms. Epidemiol Psichiatr Soc (2009) 18(4):299–310. 20170043

[36] Schreier A Wolke D Thomas K Horwood J Hollis C Gunnell D Prospective study of peer victimization in childhood and psychotic symptoms in a nonclinical population at age 12 years. Arch Gen Psychiatry (2009) 66(5):527–36. 10.1001/archgenpsychiatry.2009.23 19414712

[37] Larsson S Andreassen OA Aas M Rossberg JI Mork E Steen NE High prevalence of childhood trauma in patients with schizophrenia spectrum and affective disorder. Compr Psychiatry (2013) 54(2):123–7. 10.1016/j.comppsych.2012.06.009 22901835

[38] Matheson SL Shepherd AM Pinchbeck RM Laurens KR Carr VJ. Childhood adversity in schizophrenia: a systematic meta-analysis. Psychol Med (2013) 43(2):225–38. 10.1017/S0033291712000785 22716913

[39] Ball HA Arseneault L Taylor A Maughan B Caspi A Moffitt TE. Genetic and environmental influences on victims, bullies and bully-victims in childhood. J Child Psychol Psychiatry (2008) 49(1):104–12. 10.1111/j.1469-7610.2007.01821.x 18181884

[40] Read J Childhood adversity and psychosis: from heresy to certainty. 2 ed In: Read J Dillon J, editors. Models of Madness: Psychological, Social, and Biological Approaches to Psychosis. London: Routledge (2013). p. 249–75.

[41] Shevlin M Houston JE Dorahy MJ Adamson G. Cumulative traumas and psychosis: an analysis of the national comorbidity survey and the British Psychiatric Morbidity Survey. Schizophr Bull (2008) 34(1):193–9. 10.1093/schbul/sbm069 17586579 PMC2632373

[42] Kendler KS. Overview: a current perspective on twin studies of schizophrenia. Am J Psychiatry (1983) 140(11):1413–25. 10.1176/ajp.140.11.1413 6353948

[43] Jaffee SR Caspi A Moffitt TE Polo-Tomas M Price TS Taylor A. The limits of child effects: evidence for genetically mediated child effects on corporal punishment but not on physical maltreatment. Dev Psychol (2004) 40(6):1047–58. 10.1037/0012-1649.40.6.1047 15535755

[44] Jaffee SR Caspi A Moffitt TE Taylor A. Physical maltreatment victim to antisocial child: evidence of an environmentally mediated process. J Abnorm Psychol (2004) 113(1):44–55. 10.1037/0021-843X.113.1.44 14992656

[45] Shakoor S McGuire P Cardno AG Freeman D Plomin R Ronald A. A shared genetic propensity underlies experiences of bullying victimization in late childhood and self-rated paranoid thinking in adolescence. Schizophr Bull (2015) 41(3):754–63. 10.1093/schbul/sbu142 25323579 PMC4393686

[46] Bornovalova MA Huibregtse BM Hicks BM Keyes M McGue M Iacono W. Tests of a direct effect of childhood abuse on adult borderline personality disorder traits: a longitudinal discordant twin design. J Abnorm Psychol (2013) 122(1):180–94. 10.1037/a0028328 22686871 PMC3482426

[47] Dinwiddie S Heath AC Dunne MP Bucholz KK Madden PA Slutske WS Early sexual abuse and lifetime psychopathology: a co-twin-control study. Psychol Med (2000) 30(1):41–52. 10.1017/S0033291799001373 10722174

[48] Jay Schulz-Heik R Rhee SH Silvern L Lessem JM Haberstick BC Hopfer C Investigation of genetically mediated child effects on maltreatment. Behav Genet (2009) 39(3):265–76. 10.1007/s10519-009-9261-4 19283463 PMC2693353

[49] Lau JY Eley TC. Disentangling gene-environment correlations and interactions on adolescent depressive symptoms. J Child Psychol Psychiatry (2008) 49(2):142–50. 10.1111/j.1469-7610.2007.01803.x 18211276

[50] Rooks C Veledar E Goldberg J Bremner JD Vaccarino V. Early trauma and inflammation: role of familial factors in a study of twins. Psychosom Med (2012) 74(2):146–52. 10.1097/PSY.0b013e318240a7d8 22286843 PMC3307790

[51] Silberg J Pickles A Rutter M Hewitt J Simonoff E Maes H The influence of genetic factors and life stress on depression among adolescent girls. Arch Gen Psychiatry (1999) 56(3):225–32. 10.1001/archpsyc.56.3.225 10078499

[52] Stein MB Jang KL Taylor S Vernon PA Livesley WJ. Genetic and environmental influences on trauma exposure and posttraumatic stress disorder symptoms: a twin study. Am J Psychiatry (2002) 159(10):1675–81. 10.1176/appi.ajp.159.10.1675 12359672

[53] Vinkhuyzen AA van der Sluis S de Geus EJ Boomsma DI Posthuma D Genetic influences on ‘environmental’ factors. Genes Brain Behav (2010) 9(3):276–87 10.1111/j.1601-183X.2009.00554.x 20050926

[54] Young-Wolff KC Kendler KS Ericson ML Prescott CA. Accounting for the association between childhood maltreatment and alcohol-use disorders in males: a twin study. Psychol Med (2011) 41(1):59–70. 10.1017/S0033291710000425 20346194 PMC3010204

[55] Jang KL Vernon PA Livesley WJ Stein MB Wolf H. Intra- and extra-familial influences on alcohol and drug misuse: a twin study of gene-environment correlation. Addiction (2001) 96(9):1307–18. 10.1046/j.1360-0443.2001.969130710.x 11672495

[56] Fisher RA On the probable error of a coefficient of correlation deduced from a small sample. Metron (1921) 1:3–32.

[57] Kendler KS Karkowski LM Prescott CA. Causal relationship between stressful life events and the onset of major depression. Am J Psychiatry (1999) 156(6):837–41. 10.1176/ajp.156.6.837 10360120

[58] Slutske WS Heath AC Dinwiddie SH Madden PA Bucholz KK Dunne MP Modeling genetic and environmental influences in the etiology of conduct disorder: a study of 2,682 adult twin pairs. J Abnorm Psychol (1997) 106(2):266–79. 10.1037/0021-843X.106.2.266 9131847

[59] Plomin R DeFries JC Loehlin JC Genotype-environment interaction and correlation in the analysis of human behavior. Psychol Bull (1977) 84(2):309–22 10.1037/0033-2909.84.2.309 557211

[60] Bouchard TJ Jr Lykken DT McGue M Segal NL Tellegen A Sources of human psychological differences: the Minnesota study of twins reared apart. Science (1990) 250(4978):223–8 10.1126/science.2218526 2218526

[61] Kendler KS Baker JH. Genetic influences on measures of the environment: a systematic review. Psychol Med (2007) 37(5):615–26. 10.1017/S0033291706009524 17176502

[62] Arseneault L Cannon M Fisher HL Polanczyk G Moffitt TE Caspi A. Childhood trauma and children’s emerging psychotic symptoms: a genetically sensitive longitudinal cohort study. Am J Psychiatry (2011) 168(1):65–72. 10.1176/appi.ajp.2010.10040567 20952460 PMC3536053

[63] Alemany S Goldberg X van Winkel R Gasto C Peralta V Fananas L. Childhood adversity and psychosis: examining whether the association is due to genetic confounding using a monozygotic twin differences approach. Eur Psychiatry (2013) 28(4):207–12. 10.1016/j.eurpsy.2012.03.001 22944339

[64] Valentino K Nuttall AK Comas M Borkowski JG Akai CE. Intergenerational continuity of child abuse among adolescent mothers: authoritarian parenting, community violence, and race. Child Maltreat (2012) 17(2):172–81. 10.1177/1077559511434945 22287568

[65] Berry K Barrowclough C Wearden A. A review of the role of adult attachment style in psychosis: unexplored issues and questions for further research. Clin Psychol Rev (2007) 27(4):458–75. 10.1016/j.cpr.2006.09.006 17258365

[66] Losoya SH Callor S Rowe DC Goldsmith HH. Origins of familial similarity in parenting: a study of twins and adoptive siblings. Dev Psychol (1997) 33(6):1012–23. 10.1037/0012-1649.33.6.1012 9383623

[67] Espelage DL De La Rue L. School bullying: its nature and ecology. Int J Adolesc Med Health (2012) 24(1):3–10. 10.1515/ijamh.2012.002 22909906

[68] Lodge DJ Grace AA. Hippocampal dysregulation of dopamine system function and the pathophysiology of schizophrenia. Trends Pharmacol Sci (2011) 32(9):507–13. 10.1016/j.tips.2011.05.001 21700346 PMC3159688

[69] Teicher MH Anderson CM Polcari A. Childhood maltreatment is associated with reduced volume in the hippocampal subfields CA3, dentate gyrus, and subiculum. Proc Natl Acad Sci USA (2012) 109(9):E563–72. 10.1073/pnas.1115396109 22331913 PMC3295326

[70] Read J Fosse R Moskowitz A Perry BD The traumagenic neurodevelopmental model of psychosis revisited. Neuropsychiatry (2014) 4(1):65–79 10.2217/npy.13.89

[71] Champagne FA Curley JP. Epigenetic mechanisms mediating the long-term effects of maternal care on development. Neurosci Biobehav Rev (2009) 33(4):593–600. 10.1016/j.neubiorev.2007.10.009 18430469

[72] Tyrka AR Price LH Marsit C Walters OC Carpenter LL. Childhood adversity and epigenetic modulation of the leukocyte glucocorticoid receptor: preliminary findings in healthy adults. PLoS One (2012) 7(1):e30148. 10.1371/journal.pone.0030148 22295073 PMC3266256

[73] Caudal D Jay TM Godsil BP. Behavioral stress induces regionally-distinct shifts of brain mineralocorticoid and glucocorticoid receptor levels. Front Behav Neurosci (2014) 8:19. 10.3389/fnbeh.2014.00019 24523684 PMC3905199

[74] Galhardo RS Hastings PJ Rosenberg SM. Mutation as a stress response and the regulation of evolvability. Crit Rev Biochem Mol Biol (2007) 42(5):399–435. 10.1080/10409230701648502 17917874 PMC3319127

[75] Hicks BM South SC Dirago AC Iacono WG McGue M. Environmental adversity and increasing genetic risk for externalizing disorders. Arch Gen Psychiatry (2009) 66(6):640–8. 10.1001/archgenpsychiatry.2008.554 19487629 PMC2717707

[76] Gillespie NA Zhu G Neale MC Heath AC Martin NG. Direction of causation modeling between cross-sectional measures of parenting and psychological distress in female twins. Behav Genet (2003) 33(4):383–96. 10.1023/A:1025365325016 14574138

[77] Harlaar N Santtila P Bjorklund J Alanko K Jern P Varjonen M Retrospective reports of parental physical affection and parenting style: a study of Finnish twins. J Fam Psychol (2008) 22(4):605–13. 10.1037/0893-3200.22.3.605 18729674

[78] Barker-Collo S Read J. Models of response to childhood sexual abuse: their implications for treatment. Trauma Violence Abuse (2003) 4(2):95–111. 10.1177/1524838002250760 14697117

[79] Alford JR Funk CL Hibbing JR. Are political orientations genetically transmitted? Am Political Sci Rev (2005) 99(02):153–67. 10.1037/a0025560 21988277

